# Interactions Between Genetic Risk and Macronutrient Intake on Obesity-Related Traits in Sri Lankan Adults

**DOI:** 10.3390/nu18182951

**Published:** 2026-09-09

**Authors:** Manahil M. Bineid, Dilki S. Perera, Zara Crosbie, Ananda Chandrasekara, Kumari M. Rathnayake, Karani Santhanakrishnan Vimaleswaran

**Affiliations:** 1 Hugh Sinclair Unit of Human Nutrition, Department of Food and Nutritional Sciences, Institute for Cardiovascular and Metabolic Research (ICMR), University of Reading, Reading RG6 6DZ, UK; m.m.a.bineid@pgr.reading.ac.uk; 2 Department of Food and Nutritional Sciences, College of Agriculture and Food Sciences, King Faisal University, Al-Ahsa 31982, Saudi Arabia; 3 Department of Nutrition and Dietetics, Faculty of Livestock, Fisheries and Nutrition, Wayamba University of Sri Lanka, Makandura 60170, Sri Lanka; dilkip@wyb.ac.lk (D.S.P.); ananda.ch@wyb.ac.lk (A.C.); 4 Human Nutrition, School of Agriculture and Food Science, University College Dublin, D04 V1W8 Dublin, Ireland; z.crosbie@reading.ac.uk; 5 The Institute for Food, Nutrition and Health (IFNH), University of Reading, Reading RG6 6AH, UK

**Keywords:** genetic risk score, carbohydrates, fat, gene–diet interaction, obesity, Sri Lanka

## Abstract

Background/Objectives: In Sri Lanka, where obesity is increasingly prevalent, evidence on gene–diet interactions involving macronutrient intake and composition remains limited. We therefore examined these interactions on obesity-related traits. Methods: This cross-sectional analysis included 398 adults (mean age, 42.5 ± 8.3 years; 60.8% men). Anthropometric measurements defined increased body mass index (BMI) and waist circumference (WC). Dietary macronutrient intake was assessed with a validated tool, and carbohydrate/fat intake patterns were determined based on study population-specific macronutrient cutoffs and interpreted as relative dietary patterns rather than clinically defined diets. A genetic risk score (GRS) was constructed using three single-nucleotide polymorphisms (*FTO* rs8050136, rs1588413, and *TCF7L2* rs7903146). Associations and interaction effects were examined after adjustment for relevant confounders. Results: The GRS was positively associated with increased WC (*p* = 0.032). Carbohydrate/fat intake pattern was associated with increased BMI (*p* = 0.044) and WC (*p* = 0.021), with lower odds in the high-carbohydrate/low-fat and other combination patterns than in the low-carbohydrate/high-fat reference group. Significant GRS–carbohydrate interactions were observed for increased BMI (*p*_interaction_ = 0.008) and WC (*p*_interaction_ = 0.033), with higher odds among high-GRS individuals in the low-carbohydrate group. A GRS–fat interaction was also observed for increased BMI (*p*_interaction_ = 0.03), with significantly higher odds among high-GRS individuals in the high-fat group. Within the low-carbohydrate/high-fat pattern, high-GRS individuals had higher odds of increased BMI (OR= 3.1, *p* = 0.014). Conclusions: Dietary carbohydrate and fat intakes may influence the association between genetic susceptibility and obesity in Sri Lankan adults, particularly under a relatively low-carbohydrate/high-fat pattern.

## 1. Introduction

Obesity is an increasing public health concern in South Asia, where its rising prevalence contributes significantly to the burden of cardiometabolic diseases [1,2]. Recent global estimates indicate that South Asia is among the regions projected to experience the largest relative increase in overweight and obesity by 2050, with particularly pronounced increases in both men and women [1]. Within this context, Sri Lanka, a lower-middle-income country undergoing rapid socioeconomic and nutritional transition, has experienced a steady rise in adiposity-related conditions over the past two decades. [3,4]. South Asian populations, including Sri Lankans, exhibit a higher propensity for central adiposity and related metabolic risks at lower body mass index (BMI) and waist circumference (WC) thresholds than other ethnic groups [5,6], supporting population-specific assessment of obesity-related traits [7]. Obesity is multifactorial, reflecting interactions between genetic susceptibility and environmental exposures, particularly diet [8].

Several epidemiological studies have reported an inverse association between carbohydrate intake and the risk of overweight/obesity and a positive association between fat intake and obesity risk [9,10,11]. Beyond absolute macronutrient intake, the relative balance between carbohydrates and fat may also be relevant to body weight- and adiposity-related outcomes, potentially through metabolic mechanisms that affect energy allocation and storage [12,13,14,15]. Although Sri Lanka is undergoing a gradual nutrition transition [16,17], adult diets remain relatively high in carbohydrates, contributing approximately 70–73% of total energy intake (TEI), with lower contributions from fat and protein [18,19]. These dietary characteristics may be particularly relevant in individuals with greater genetic susceptibility, as emerging evidence suggests that high-fat dietary patterns may amplify genetic risk of obesity [20].

Despite the growing global interest in gene–diet interactions, evidence from South Asian populations remains limited [21]. Given substantial differences in genetic architecture, habitual dietary patterns, and metabolic profiles, findings from Western cohorts may not translate directly to South Asian populations. In Sri Lanka, although genetic and lifestyle factors have been examined independently, evidence integrating both domains remains sparse, with only a few studies reporting limited gene–diet interactions associated with cardiometabolic outcomes [22,23]. Accordingly, this study examined genetic susceptibility, dietary factors and their interactions on obesity-related traits in a Sri Lankan adult cohort, with particular focus on macronutrient and carbohydrate- and fat-intake patterns. To capture cumulative genetic susceptibility, this study constructed a three-SNP genetic risk score (GRS) comprising three single-nucleotide polymorphisms (SNPs) from fat mass and obesity-associated (*FTO*) and transcription factor 7-like 2 (*TCF7L2*) genes, providing a more powerful approach for assessing genetic susceptibility and detecting gene–diet interactions [24,25]. These SNPs were selected based on previous evidence linking *FTO* variants to adiposity and *TCF7L2* rs7903146 to metabolic and adiposity-related traits, including diet-related anthropometric responses [26,27,28,29,30,31,32,33,34,35].

## 2. Materials and Methods

### 2.1. Study Design and Participants

Overall, 398 adults were included in this cross-sectional study. Volunteers who contacted the Department of Nutrition and Dietetics at Wayamba University of Sri Lanka were provided with a study outline and asked to complete a medical and lifestyle questionnaire by telephone or e-mail. Individuals who met the eligibility criteria based on questionnaire responses were invited to participate; during the study visit, detailed information about the study procedures was provided.

This study was conducted in accordance with the Declaration of Helsinki, and all procedures involving human participants were approved by the Sri Lankan Medical Association (SLMA; approval no.: 2020-11; approved on 16 October 2020). Written informed consent was obtained from all participants before enrolment. To maintain confidentiality, each participant was assigned a unique identification code that was used throughout this study, including during sample handling and data analysis. Study procedures were conducted at the Clinical Laboratory and Nutrition and Wellness Centre of the Department of Nutrition and Dietetics at Wayamba University of Sri Lanka.

Participants were non-smokers, did not consume excessive alcohol (>15 units/week; 1 unit = 125 mL), were not on a weight-reducing regimen, and were not taking nutritional supplements. The exclusion criteria included a prior diagnosis of chronic diseases (e.g., diabetes, hypertension, dyslipidaemia, cardiovascular disease, cancer, liver or renal disease, and anaemia) or use of related medications (antihypertensive, antidiabetic, lipid-lowering, or anti-inflammatory drugs). These exclusion criteria were applied to minimise the influence of factors that could independently affect vascular and metabolic outcomes, including smoking, higher alcohol intake, active weight loss, nutritional supplement use, chronic disease, and related medication use. This study employed a cross-sectional design, in which data were collected from participants at a single time point.

### 2.2. Anthropometric and Biochemical Measurements

Anthropometric and body composition measurements were obtained with participants wearing light clothing and without shoes or metal objects. Height was measured to the nearest 0.1 cm using a stadiometer, with participants standing upright, facing forward, and maintaining the Frankfort plane. Body weight was assessed using a bioelectrical impedance analyser (TANITA MC-780, TANITA Corporation, Tokyo, Japan), and a standard 0.5 kg deduction was applied for light clothing. Waist circumference was measured at the midpoint between the lowest rib and the iliac crest using a non-stretchable tape (Seca, Birmingham, UK). All measurements were performed by a trained researcher in accordance with standard protocols. Venous blood samples (5 mL) were collected in EDTA vacutainer tubes, and whole blood samples were stored at −80 °C for genotyping analysis.

### 2.3. Dietary Assessment

The participants’ habitual dietary intake was assessed using a 3-day food diary that included 2 weekdays and 1 weekend day. Participants were instructed to record all food and beverages consumed, including portion sizes and any leftovers. To improve accuracy, written and verbal instructions were provided, supported by a completed sample diary. Standardised portion-size references, including the Sri Lankan Food Atlas [36] and common household measures (e.g., coconut spoons, teaspoons, tablespoons, and teacups), were used to aid estimation. Before data entry, all household measurements were converted to grams using standard food portion-size tables. Nutrient intake was then analysed using FoodBase 2000 (Institute of Brain Chemistry, London, UK), which was specifically developed for nutrient analysis of Sri Lankan foods.

### 2.4. Genotyping and SNP Selection

Peripheral venous blood samples were collected for DNA analysis and were transported to the United Kingdom (UK) on dry ice under controlled temperature conditions. Genomic DNA was isolated from 5 mL of the whole blood obtained from each participant. Genotyping of the selected SNPs was performed by LGC Biosearch Technologies™ (London, UK; https://www.biosearchtech.com/services/genotyping-services, accessed on 15 February 2026) using the Kompetitive Allele-Specific PCR (KASP^®^) assay(LGC Biosearch Technologies, Hoddesdon, UK).

This study focused on three SNPs: *FTO* rs8050136 and rs1588413 [26,27,28,29] and *TCF7L2* rs7903146 [30,31,32,33,34,35]. These SNPs were selected based on previous evidence linking the *FTO* variants to obesity- and adiposity-related traits and *TCF7L2* rs7903146 to metabolic and adiposity-related phenotypes, including diet-related anthropometric responses [26,27,28,29,30,31,32,33,34,35]. The SNPs were combined into a GRS to assess their cumulative contribution to genetic susceptibility, and their inclusion was based on prior evidence rather than statistical significance in the present sample. Genotypic distributions were assessed for Hardy–Weinberg equilibrium (HWE) using the chi-square test, and all 3 loci conformed to HWE expectations (*p* > 0.05), indicating no significant deviation because of genotyping error or population substructure (Supplementary Table S1).

A metabolic GRS was constructed to quantify the cumulative burden of risk alleles across the selected SNPs. Each genotype was coded additively as 0, 1, or 2 according to the number of alleles previously associated with adverse metabolic outcomes. In the absence of effect size estimates specific to South Asian or Sri Lankan populations for these SNPs, an unweighted GRS approach was used, with equal weights assigned to each risk allele. The resulting GRS ranged from 0 to 6, indicating the total number of risk alleles carried by an individual. Given the narrow, discrete distribution of the score, participants were dichotomised at the sample median into a low-risk group (GRS < 2 risk alleles) and a high-risk group (GRS ≥ 2 risk alleles) to facilitate interpretation of the stratified gene–diet interaction analyses.

### 2.5. Definition of Variables

Macronutrient intake was expressed as the percentage of TEI derived from carbohydrates, protein, and fat. Based on the relative contribution of carbohydrates and fat to TEI, dietary patterns were categorised as high-carbohydrate/low-fat (HC/LF) (carbohydrates > 65% of TEI and fat < 20% of TEI), low-carbohydrate/high-fat (LC/HF) (carbohydrates < 60% of TEI and fat > 25% of TEI), or other patterns. These cutoffs were derived from the macronutrient intake distribution within the study population and were intended to reflect relative differences in dietary composition rather than clinically defined low-carbohydrate or high-fat diets. The “other” category included all remaining combinations of macronutrient intake that did not meet the criteria for the defined patterns.

BMI was calculated as weight in kilograms divided by the square of height in metres (kg/m^2^). Increased BMI was defined as BMI ≥ 23 kg/m^2^, consistent with recommendations for Asian populations, as this cutoff reflects an increased risk of cardiometabolic conditions [37]. Increased BMI was designated as the primary outcome, while increased WC was considered the secondary outcome. WC was measured using standardised procedures; increased WC was defined as ≥90 cm in men and ≥80 cm in women, based on ethnicity-specific thresholds for Asian populations [38]. All anthropometric measurements, including BMI and WC, were performed in accordance with standard protocols.

### 2.6. Statistical Analysis

The sample size of the parent study was calculated to detect a 0.2 standard deviation (SD) difference in pulse wave velocity (PWV), its primary outcome, as previously described [39]. As previously reported effect-size estimates for comparable gene–diet interactions on obesity-related traits were unavailable for the Sri Lankan population, a separate a priori sample size calculation was not performed for the present analyses.

Data were screened for completeness, plausibility, and internal consistency before analysis. Statistical analyses were performed using SPSS (version 29; SPSS Inc., Chicago, IL, USA). Allele frequencies were calculated by direct counting. HWE was assessed for each SNP using a chi-square goodness-of-fit test. No selected SNP showed a significant deviation from HWE (*p* > 0.05), and all SNPs had a minor allele frequency greater than 5% (Supplementary Table S1). Normality of variables was assessed using the Shapiro–Wilk test. Variables deviating from normality were log-transformed before analysis to meet the assumptions of normality for descriptive analyses and comparisons of continuous variables [40,41]. For association and interaction analyses, increased BMI and WC were analysed as categorical variables and dichotomised using established cutoff values based on the original, non-transformed measurements.

Descriptive statistics for continuous variables were presented as means and SDs, whereas categorical variables were presented as percentages. Differences in continuous variables between male and female participants were assessed using an independent-samples *t*-test on log-transformed data. Categorical variables between groups were compared using the chi-square test. Linear regression was used to examine the association between individual SNPs and continuous obesity-related outcomes after adjustment for age and sex (Supplementary Table S2). Associations between the GRS, carbohydrate and fat intake patterns, and increased BMI and WC were assessed using multivariable logistic regression. All logistic regression models were adjusted for age and sex, selected based on their established associations with obesity-related traits, while models evaluating associations with carbohydrate and fat intake patterns were further adjusted for TEI. Given the modest sample size and inclusion of interaction terms, a parsimonious adjustment set was used to limit model complexity. Interactions between the GRS and macronutrient intake, expressed as percentages of TEI (carbohydrate, fat, and protein), were examined by adding interaction terms (GRS × macronutrient intake) to the multivariable logistic regression models. All interaction models were adjusted for age, sex, and TEI. Statistically significant interactions were further explored using stratified analyses by macronutrient intake category, with adjusted odds ratios estimated within each stratum. Odds ratios (ORs) and 95% confidence intervals (CIs) were determined. Additionally, linear regression models were fitted with continuous BMI and WC as outcomes to assess the robustness of the findings. All statistical tests were two-sided, with a nominal significance threshold of *p* < 0.05. No formal adjustment for multiple comparisons was applied; therefore, the interaction findings were considered exploratory and interpreted cautiously.

## 3. Results

### 3.1. Baseline Characteristics of Study Participants

This study included 398 participants (mean age, 42.52 ± 8.3 years). Men and women accounted for 60.8% and 39.2% of the cohort, respectively. Participant characteristics are summarised in Table 1. The mean BMI of the study population was 24.34 ± 3.74 kg/m^2^, and the mean WC was 86.11 ± 9.99 cm. Comparisons between male and female participants showed significant differences in weight and WC. Significant between-sex differences were also observed for TEI and the proportions of carbohydrate, protein, and fat intake (% TEI) (all *p* < 0.001). The overall prevalence of increased BMI was 63.6%, with similar proportions in men (63.2%) and women (64.1%) and no significant difference by sex (*p* = 0.85). In contrast, the prevalence of increased WC was 52.8%, with a significantly higher proportion in women (65.4%) than in men (44.6%) (*p* < 0.001). Participants were further categorised by GRS: 41.5% were classified as having a low GRS (<2 risk alleles) and 58.5% as having a high GRS (≥2 risk alleles). No significant differences in GRS distribution were observed between the groups. Participant characteristics according to GRS group are presented in Supplementary Table S3.

### 3.2. Associations Between Carbohydrate–Fat Intake Patterns and Obesity-Related Outcomes

**Figure 1:**
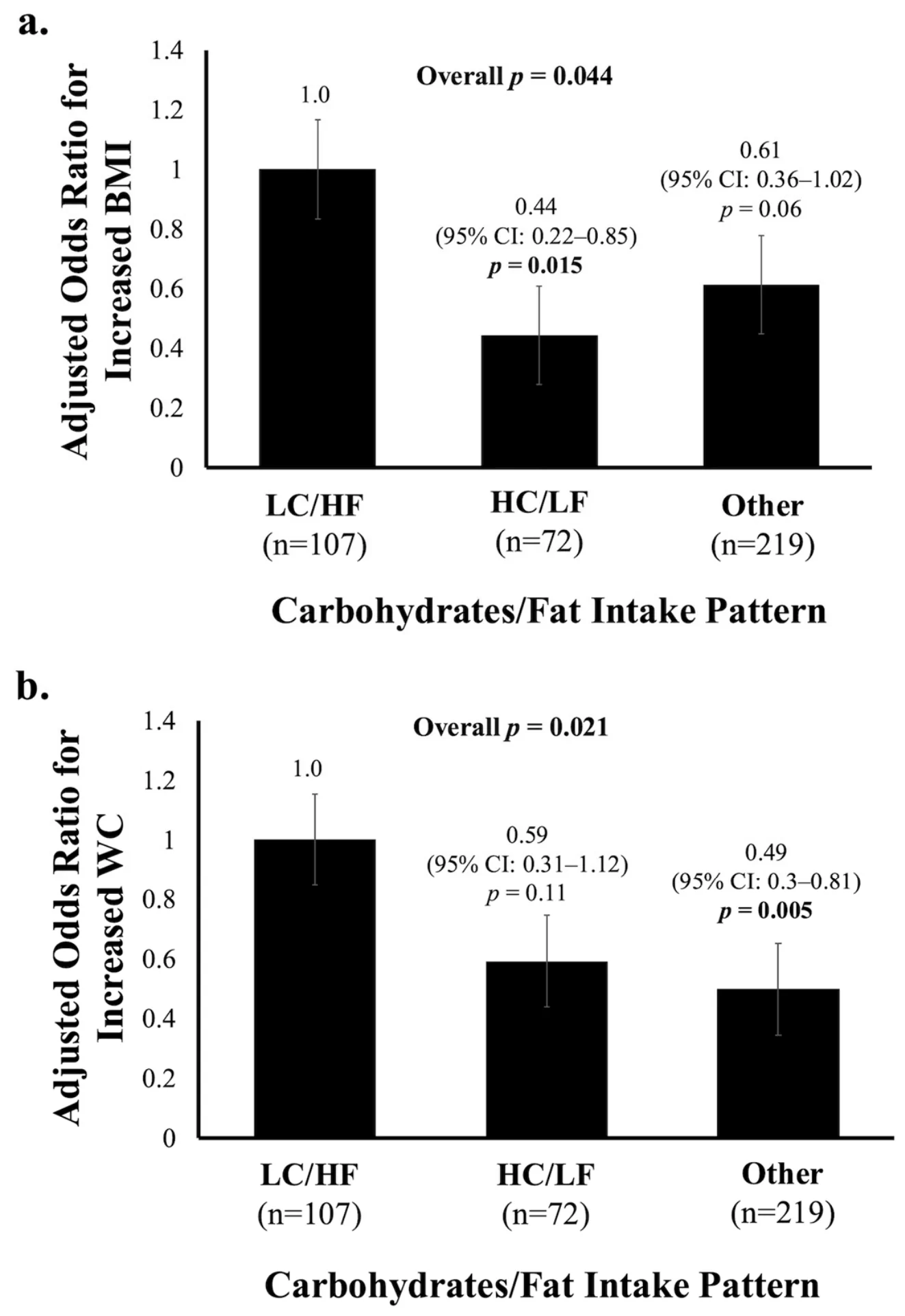
Adjusted odds ratios for increased BMI and WC across carbohydrate/fat intake patterns. Odds ratios (ORs) and 95% confidence intervals derived from a multivariable logistic regression model adjusted for age, sex, and total energy intake. (**a**) Increased BMI. (**b**) Increased WC. Abbreviations: BMI, body mass index; WC, waist circumference; LC/HF, low-carbohydrate/high-fat (carbohydrate < 60% and fat > 25%); HC/LF, high-carbohydrate/low-fat (carbohydrate > 65% and fat < 20%); other refers to other intake patterns.

Associations between carbohydrate- and fat-based dietary patterns and obesity traits were evaluated using multivariable logistic regression models adjusted for age, sex, and TEI, with the LC/HF pattern as the reference category (Table 2). The carbohydrate–fat dietary pattern was significantly associated with increased BMI (*p* = 0.044). In pattern-specific analyses, participants with the HC/LF pattern had significantly lower odds of elevated BMI compared with those with the LC/HF pattern (*p* = 0.015) (Figure 1a). A significant association was also observed between the carbohydrate–fat dietary pattern and increased WC (*p* = 0.021). In stratified comparisons, participants in the other pattern group had significantly lower odds of elevated WC than those in the LC/HF reference group (*p* = 0.005) (Figure 1b).

### 3.3. Associations Between 3-Snp Grs and Obesity-Related Outcomes

The GRS was significantly associated with increased WC; individuals in the high-GRS group had higher odds of increased WC than those in the low-GRS group (*p* = 0.032). In contrast, no significant associations were observed between GRS and BMI (Table 2).

### 3.4. Interaction Between the 3-Snp Grs and Macronutrient Intake

**Figure 2:**
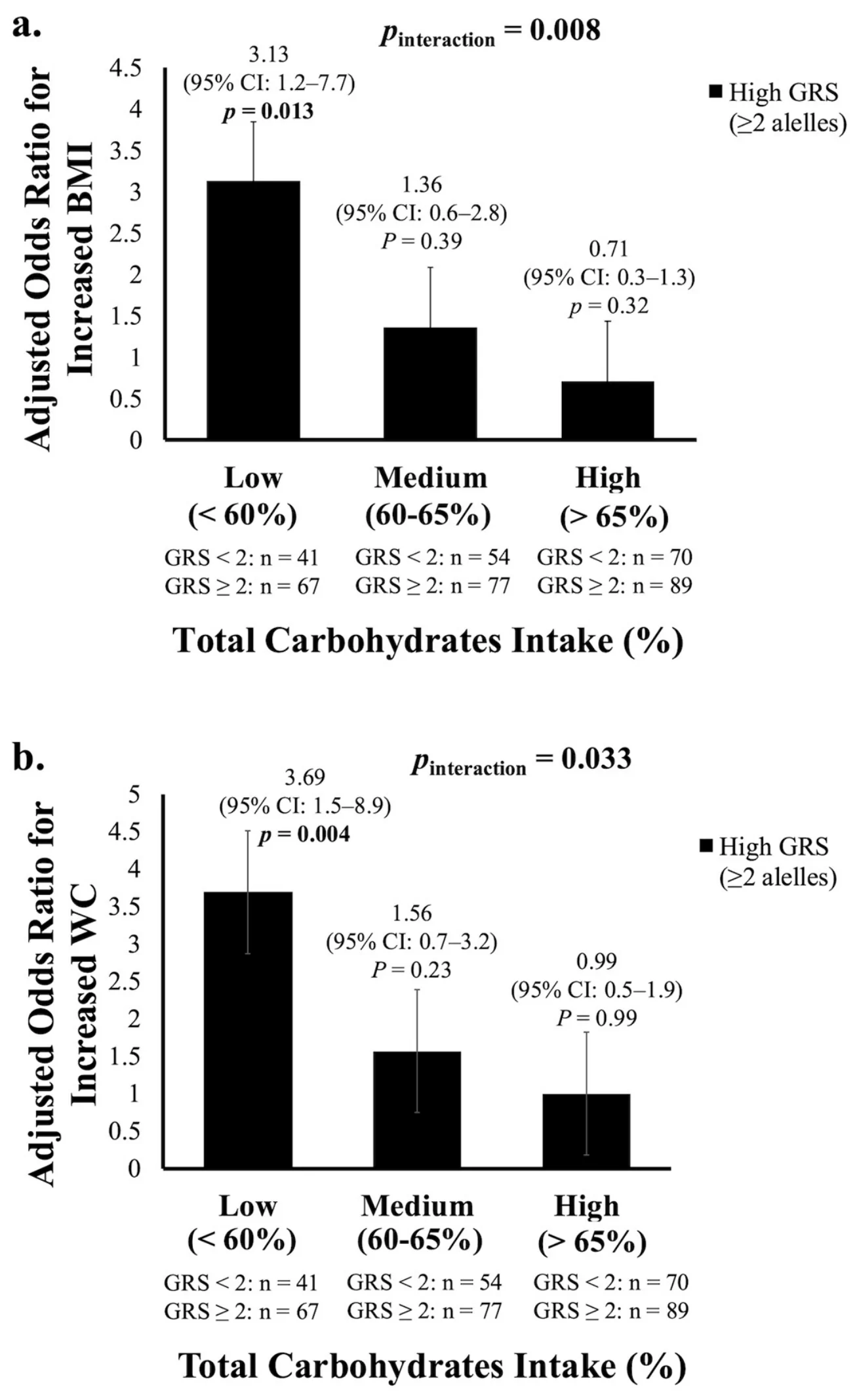
Interaction between the GRS and carbohydrate intake (%) on increased BMI and WC. Adjusted odds ratios (ORs) and 95% confidence intervals for increased BMI and WC according to carbohydrate intake (%) categories, stratified by 3-SNP GRS using multivariable logistic regression models adjusted for age, sex, and total energy intake. (**a**) Increased BMI. (**b**) Increased WC. Abbreviations: GRS, genetic risk score; BMI, body mass index; WC, waist circumference.

Significant interactions were observed between the 3-SNP GRS and macronutrient intake (expressed as a percentage of TEI) for adiposity-related markers. These interaction analyses were conducted using multivariable logistic regression models adjusted for age, sex, and TEI (Table 3). A significant interaction was identified between the GRS and carbohydrate intake (%) with respect to the risk of increased BMI (*p*_interaction_ = 0.008). In stratified analyses, within the low-carbohydrate intake group (<60% of TEI), individuals with a high-risk GRS (≥2 risk alleles) had significantly higher odds of increased BMI than those in the low-risk GRS group (*p* = 0.013) (Figure 2a). A similar interaction was observed between the GRS and carbohydrate intake (%) for the risk of increased WC (*p*_interaction_ = 0.033). As shown in Figure 2b, individuals with a high-risk GRS in the low-carbohydrate intake group had significantly higher odds of increased WC compared with those in the low GRS group (*p* = 0.004).

**Figure 3:**
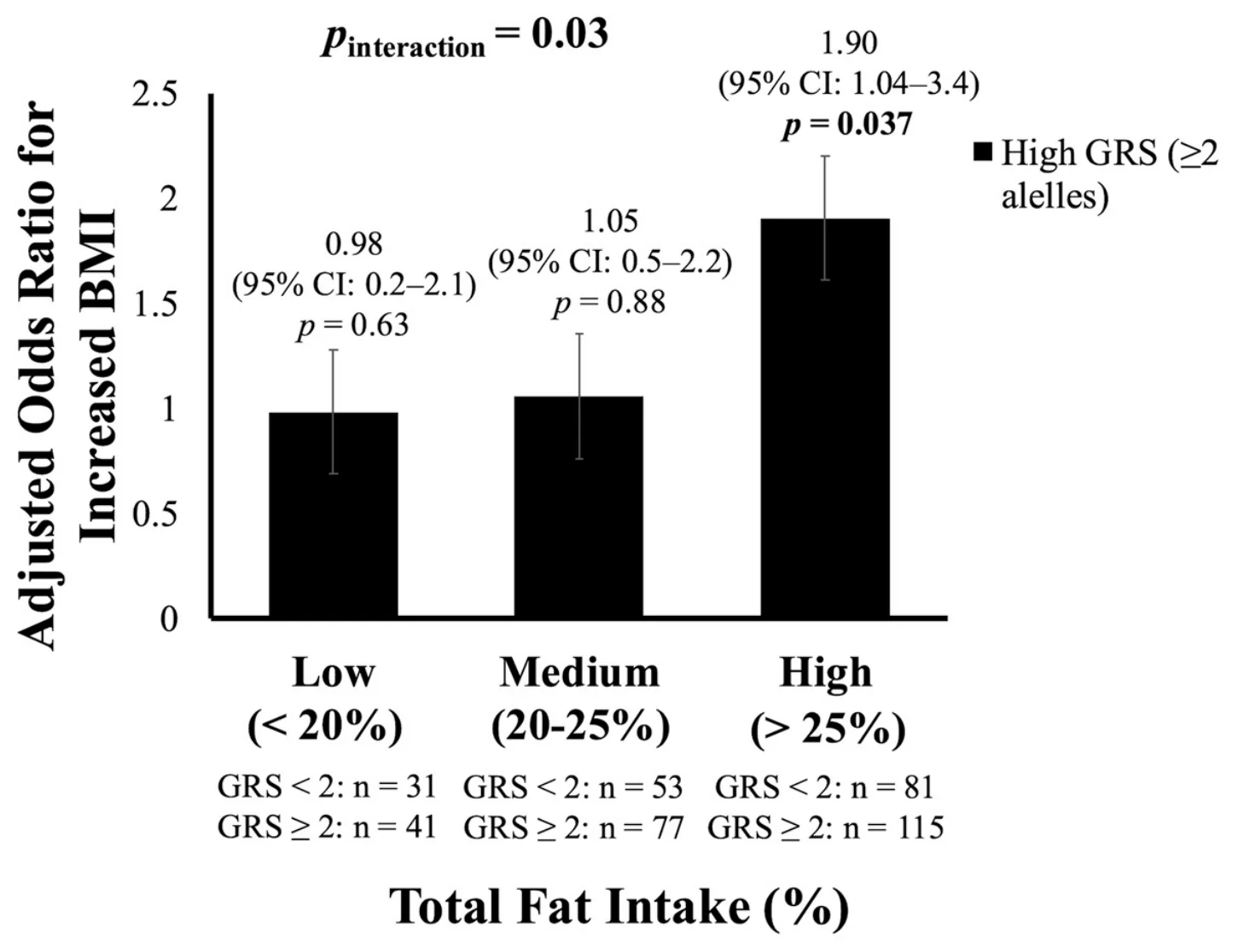
Interaction between the GRS and fat intake (%) on increased BMI. Adjusted odds ratios (ORs) and 95% confidence intervals for increased BMI according to fat intake (%) categories, stratified by 3-SNP GRS. Models were adjusted for age, sex, and total energy intake. Abbreviations: GRS, genetic risk score; BMI, body mass index.

In addition, a significant interaction was detected between the GRS and fat intake (%) with respect to the risk of increased BMI (*p*_interaction_ = 0.03). Stratified analyses showed that, among participants in the high-fat intake group (>25% TEI), those with a high-risk GRS had significantly higher odds of increased BMI than those in the low-risk GRS group (*p* = 0.037) (Figure 3). No significant differences in the odds of increased BMI were observed between the GRS groups within the low- and medium-fat intake categories.

These findings were consistent with the linear regression models using continuous outcomes, in which significant interactions were observed between the GRS and carbohydrate and fat intakes (%) for BMI and WC (all *p*_interaction_ < 0.05), whereas no significant interaction was observed with protein intake (Supplementary Table S4).

### 3.5. Differences in Genetic Risk Associations Across Carbohydrate/Fat Intake Patterns

**Figure 4:**
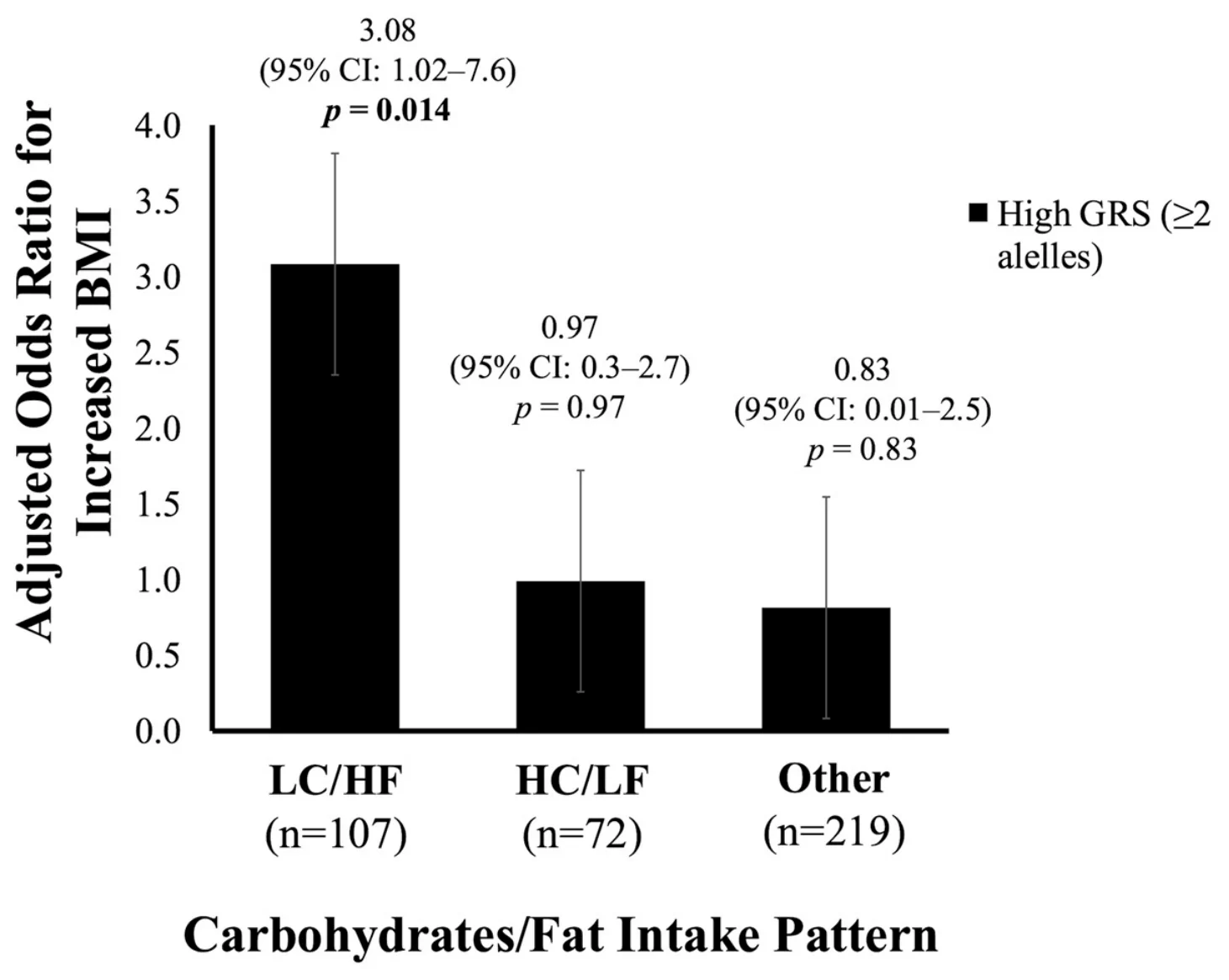
Differences in genetic risk associations with increased BMI across carbohydrate/fat intake patterns. Adjusted odds ratios (ORs) and 95% confidence intervals for increased BMI across carbohydrate/fat intake patterns, stratified by 3-SNP GRS, were estimated using multivariable logistic regression models adjusted for age, sex, and total energy intake. Abbreviations: GRS, genetic risk score; BMI, body mass index; LC/HF, low-carbohydrate/high-fat (carbohydrate < 60% and fat > 25%); HC/LF, high-carbohydrate/low-fat (carbohydrate > 65% and fat < 20%); other refers to other intake patterns.

To further characterise the observed gene–diet interaction trends involving carbohydrate and fat intake, differences in the adjusted odds ratios for increased BMI between the low- and high-risk GRS groups were examined across carbohydrate- and fat-intake patterns. Within the LC/HF pattern group, individuals carrying ≥ 2 risk alleles had significantly higher adjusted odds of increased BMI than those carrying < 2 risk alleles (OR = 3.1, *p* = 0.014), after adjustment for age, sex, and TEI. No significant differences in the odds of increased BMI were observed between the GRS groups for the HC/LF or other patterns (Figure 4).

## 4. Discussion

The current findings provide population-specific evidence of interactions between genetic risk and macronutrient intake on obesity-related traits among Sri Lankan adults. Significant associations were observed between the metabolic GRS and central obesity (WC) and between carbohydrate–fat intake patterns and adiposity measures (BMI and WC). GRS–carbohydrate interactions were observed for increased BMI and WC and a GRS–fat interaction for increased BMI, whereas no interaction was observed with protein intake. Carbohydrate/fat intake patterns were also associated with increased BMI and WC. Stratified analyses indicated stronger genetic associations at lower-carbohydrate, higher-fat dietary intake and within the relative LC/HF pattern. These findings suggest that dietary carbohydrate and fat intake may be relevant to the association between genetic susceptibility and obesity in this population.

Although none of the individual SNPs was significantly associated with obesity-related traits, the combined GRS was associated with higher WC, potentially reflecting the cumulative contribution of SNPs with modest individual effects. This finding is consistent with evidence supporting a role for *FTO* variants in obesity susceptibility and central adiposity across South and Southeast Asian populations [42,43,44,45,46]. Associations between central obesity and related traits have also been reported across Asian cohorts [45,46], as well as the proposed involvement of *FTO* in body weight regulation and energy homeostasis [42,47]. In contrast, evidence linking *TCF7L2* to obesity is less consistent, although *TCF7L2* has a well-established role in glucose metabolism and insulin function [48]. *TCF7L2* rs7903146 was included in the GRS based on its broader metabolic relevance and previous evidence linking the variant to adiposity measures and macronutrient-dependent anthropometric responses [34,35], while associations with adiposity traits remain inconsistent across studies [49,50,51].

In the present analysis, carbohydrate–fat intake patterns were significantly associated with both BMI and WC, consistent with evidence that macronutrient composition can influence body-weight regulation and adiposity-related outcomes [9,52]. Several studies have also reported inverse associations between carbohydrate intake and obesity indicators, and positive associations between fat intake and obesity risk [9,10,53]. In a cohort of middle-aged French men, carbohydrate intake was inversely associated with BMI and WC, whereas fat intake was positively associated with both outcomes, although these associations were attenuated after mutual adjustment [13]. Meta-analytic evidence indicates that reductions in total fat intake are associated with modest but significant decreases in body weight, BMI, and WC [10]. In Asian populations, replacing 5% of carbohydrate-derived energy with fat has been associated with increased overweight and obesity risk [9], while lower-carbohydrate and higher-fat intake patterns have been linked to higher body weight and WC [54]. Interventional evidence similarly suggests that low-fat, high-complex-carbohydrate diets may reduce abdominal obesity compared with higher-fat approaches [55]. Nevertheless, findings across studies remain inconsistent [10,56], and much of the existing evidence is derived from Western populations, which may limit its applicability in South Asian settings [57]. More recent South Asian evidence has associated higher carbohydrate intake with greater risks of general and abdominal obesity [58], while meta-analytic evidence on carbohydrate intake and obesity remains inconclusive [59]. These findings should therefore be interpreted within the context of Sri Lankan diets, where carbohydrates contribute over 70% of total energy and fat less than 20% [18], alongside an ongoing nutritional transition [17,60].

Emerging evidence suggests that responses to macronutrient composition may vary with genetic susceptibility [61,62]. The observed GRS–carbohydrate interactions were characterised by stronger genetic associations at lower carbohydrate and higher fat intake. Because the LC/HF category was defined relative to the study population, it reflected comparatively lower carbohydrate and higher fat intake within a generally carbohydrate-rich population rather than a clinically defined low-carbohydrate diet; the stronger genetic association may therefore reflect relative macronutrient balance rather than carbohydrate restriction itself. Previous studies from Sri Lanka have also reported gene–carbohydrate interactions associated with central obesity [23]. Consistent findings have been reported for *FTO* variation in other populations [63,64,65]. In Polish adults, interactions between *FTO* rs8050136 genotypes and macronutrient intake showed that higher carbohydrate and lower fat intake were associated with a more favourable body composition [63]. Similarly, a large population-based study found stronger *FTO* associations with BMI and obesity under high-fat, low-carbohydrate intake [65]. Findings from the UK Biobank similarly showed a significant interaction between the obesity GRS and total fat intake, with stronger genetic associations with BMI among individuals consuming high-fat diets [66]. Evidence for *TCF7L2*–diet interactions with obesity-related traits is more limited. In randomised weight-loss trials, *TCF7L2* rs7903146 interacted with dietary fat–carbohydrate composition in relation to changes in body weight and WC [35], while rs12255372 interacted with dietary fat for short-term changes in BMI and fat mass, although these effects were not sustained at 24 months and no interaction with carbohydrate intake was observed [67].

Gene–diet interactions may arise through biological pathways by which dietary macronutrient exposure influences genetic susceptibility. Dietary fat may contribute to adiposity through its high energy density and efficient storage, while fatty acid composition can influence lipid metabolism and insulin sensitivity [68]. Higher-fat foods may also promote greater energy intake through their energy density and eating characteristics [69,70]. At the genetic level, *FTO* variants are implicated in appetite and satiety regulation, with risk alleles associated with higher energy intake and altered hormonal signalling [71]. These variants have also been linked to greater fat intake, suggesting a behavioural pathway through which high-fat dietary environments may interact with genetic predispositions. Experimental evidence indicates that macronutrient composition may modify metabolic responses according to *FTO* genotype [72]. Similarly, *TCF7L2* plays a role in adipocyte differentiation and adipose tissue development through the Wnt signalling pathway, with altered *TCF7L2* activity affecting adipogenesis and fat mass expansion [73]. Observational and intervention studies suggest that *TCF7L2* risk variants are associated with differential adiposity responses across dietary fat intake levels, with greater reductions in BMI and body fat reported under lower-fat conditions [67,74]. These mechanisms may contribute to inter-individual differences in adiposity.

Key strengths of this study include its focus on Sri Lankan adults, an understudied group in nutrigenetic research, and the use of a multi-SNP GRS to assess cumulative genetic susceptibility [25]. Evaluating both macronutrient intake and carbohydrate–fat composition enabled assessment of dietary balance relative to genetic risk. However, this study had some limitations. The cross-sectional design limits causal inference and does not allow assessment of temporal relationships [75], while dietary intake was self-reported at a single time point and may not fully capture long-term habitual intake [76]. The dietary pattern categories were derived from the study population and therefore do not represent clinically defined low-carbohydrate or high-fat diets. In addition, the “other” category may obscure differences between individual carbohydrate–fat combinations. Furthermore, the GRS is based on a limited number of SNPs and may not reflect broader polygenic susceptibility. The sample size was determined for the primary outcome of the parent study rather than specifically for the present gene–diet interaction analyses. Stratification by genetic risk and dietary intake resulted in relatively small subgroups, which may have limited the detection of modest interaction effects and the precision of some estimates. Residual confounding from unmeasured lifestyle and sociodemographic factors cannot be excluded. Accordingly, the interaction findings should be regarded as exploratory and require confirmation in larger independent studies. Finally, volunteer recruitment and restrictive eligibility criteria may limit the sample’s representativeness and its generalisability to the broader Sri Lankan population.

By examining gene–diet interactions in a Sri Lankan population, this study contributes population-specific evidence to the limited literature on South Asian nutrigenetics and highlights the relevance of considering both genetic susceptibility and macronutrient composition in obesity-related research. Future research should consider macronutrient quality and food sources alongside quantity, because of their differential associations with disease risk [58]. In addition, emerging evidence indicates that the gut microbiota may modulate host metabolic responses to macronutrient composition, highlighting the value of integrating microbiome-related measures in future nutrigenetic studies [61]. Prospective and intervention studies using expanded polygenic risk scores and objective dietary biomarkers are needed to better establish the robustness and potential causality of observed gene–diet interactions. Replication across larger, more diverse South Asian cohorts will be essential to strengthen generalisability and inform precision nutrition approaches.

## 5. Conclusions

This study identified interaction effects between metabolic GRS and dietary carbohydrate and fat intake on obesity-related markers in Sri Lankan adults. These findings suggest that the association between genetic risk and obesity may vary across dietary contexts, particularly in how fat and carbohydrates contribute to TEI. Although a causal inference cannot be established, the observed patterns suggest that the association between genetic susceptibility and obesity-related traits was strongest among individuals consuming a relatively low-carbohydrate/high-fat diet. Together, these results highlight the potential value of integrating genetic risk with dietary assessment to inform population-relevant personalised nutrition approaches. They also support dietary pattern modification as a potential means of reducing obesity-related risk, pending confirmation in prospective or intervention studies among individuals with higher genetic susceptibility.

## Figures and Tables

**Table 1:** Baseline characteristics of the study population (*n* = 398).

	*n*	Mean	SD	Men			Women			*p*-Value
				*n*	Mean	SD	*n*	Mean	SD	
Age	398	42.52	8.3	242	43.07	8.23	156	41.65	8.35	0.084 *
Weight (kg)	398	64.12	12.07	242	67.91	11	156	58.25	11.31	**1.52 × 10^−15^** *
WC (cm)	398	86.11	9.99	242	88.38	9.27	156	82.58	10.07	**1.71 × 10^−8^** *
BMI	398	24.34	3.74	242	24.26	3.38	156	24.46	4.25	0.88 *
Energy (kcal)	398	1898.61	569.54	242	2136.08	528.91	156	1530.22	413.80	1.14 × 10^−31^ *
Carbohydrate (energy%)	398	63.44	6.1	242	64.85	6.31	156	61.24	5.05	**8.61 × 10^−10^**
Fat (energy %)	398	24.95	5.48	242	23.68	5.51	156	26.93	4.81	**3.58 × 10^−9^**
Protein (energy %)	398	12.28	5.4	242	11.34	2.53	156	13.69	7.84	**4.46 × 10^−4^**
Fibre (g)	398	8.81	3.57	242	8.90	3.54	156	8.69	3.61	0.135 *
3-SNP GRS (%)	398			242			156			0.782 ^†^
Low < 2	165	41.5		99	40.9		66	42.3		
High ≥ 2	233	58.5		143	59.1		90	57.7		
Increased BMI (%)	398	63.6		153	63.2		100	64.1		0.859 ^†^
Increased WC (%)	398	52.8		108	44.6		102	65.4		**5.13 × 10^−5 †^**

Data are presented as mean and SD for continuous variables and percentages for categorical variables. *p*-values for continuous variables were derived using an independent-sample *t*-test. * Analyses were conducted on log-transformed variables. ^†^ *p*-values for categorical variables were obtained by a chi-square test. Values in bold represent statistical significance (*p* < 0.05). Abbreviations: WC, waist circumference; BMI, body mass index; GRS, genetic risk score.

**Table 2:** Association of 3-SNP GRS and carbohydrate/fat patterns with obesity-related outcomes.

Exposure	Category	Increased BMI			Increased WC		
		OR	95% CI	*p*-Value	OR	95% CI	*p*-Value
GRS	GRS < 2	1 (ref.)		0.234	1 (ref.)		**0.032**
	GRS ≥ 2	1.29	0.85–1.95		1.57	1.04–2.37	
Carbohydrate/fat pattern	LC/HF	1 (ref.)		0.044 *	1 (ref.)		0.021 *
	HC/LF	0.44	0.22–0.85		0.59	0.31–1.12	
	Other	0.61	0.36–1.02		0.49	0.30–0.81	

*p*-Values were obtained using a multivariable logistic regression model. Model adjusted for age and sex. * Model was additionally adjusted for total energy intake. Values in bold represent statistical significance (*p* < 0.05). Abbreviations: GRS, genetic risk score; BMI, body mass index; WC, waist circumference; LC/HF, low-carbohydrate/high-fat (carbohydrate < 60% and fat > 25%); HC/LF, high-carbohydrate/low-fat (carbohydrate > 65% and fat < 20%); other refers to other intake patterns. Increased WC (≥90 cm for men and ≥80 cm for women) and increased BMI (≥23 kg/m^2^).

**Table 3:** Interaction between 3-SNP GRS with macronutrient energy (%) on obesity-related outcomes.

Obesity Outcomes	GRS×Fat (%)			GRS × Carbohydrates (%)			GRS × Protein (%)		
	OR	95% CI	*p*-Value	OR	95% CI	*p*-Value	OR	95% CI	*p*-Value
Increased BMI	1.09	1.00–1.18	**0.030**	0.90	0.84–0.97	**0.008**	0.97	0.90–1.05	0.52
Increased WC	1.05	0.94–1.17	0.34	0.92	0.86–0.99	**0.033**	1.00	0.92–1.08	0.92

*p*_interaction_ values were obtained using a multivariable logistic regression. Model adjusted for age, sex, and total energy intake. Values in bold represent statistical significance (*p* < 0.05). Abbreviations: BMI, body mass index; WC, waist circumference. Increased WC (≥90 cm for men and ≥80 cm for women), increased BMI (≥23 kg/m^2^).

## Data Availability

The original contributions presented in this study are included in this manuscript. Further inquiries can be directed to the corresponding author.

## Supplementary Materials

The following supporting information can be downloaded at https://www.mdpi.com/article/10.3390/nu18182951/s1, Table S1: Genotypes and allele frequency of selected SNPs; Table S2: Associations of *FTO* rs8050136 and rs1588413 and *TCF7L2* SNP rs7903146 with obesity traits (additive model); Table S3: Baseline characteristics of the study population by GRS groups (*n* = 398); Table S4: Linear regression models examining GRS–macronutrient interactions on continuous obesity-related outcomes.

## Abbreviations

The following abbreviations are used in this manuscript:

BMI	Body mass index
WC	Waist circumference
SNP	Single-nucleotide polymorphism
*FTO*	Fat mass and obesity-associated gene
*TCF7L2*	Transcription factor 7-like 2
GRS	Genetic risk score
HWE	Hardy–Weinberg equilibrium
LC/HF	Low-carbohydrate/high-fat
HC/LF	High-carbohydrate/low-fat
TEI	Total energy intake
KASP	Kompetitive Allele-Specific Polymerase Chain Reaction
PCR	Polymerase chain reaction
PWV	Pulse wave velocity
SD	Standard deviation
OR	Odds ratio
CI	Confidence interval
SLMA	Sri Lankan Medical Association

## References

[1] Ng M., Gakidou E., Lo J., Abate Y.H., Abbafati C., Abbas N., Abbasian M., Abd ElHafeez S., Abdel-Rahman W.M., Abd-Elsalam S. (2025). Global, regional, and national prevalence of adult overweight and obesity, 1990–2021, with forecasts to 2050: A forecasting study for the Global Burden of Disease Study 2021. Lancet.

[2] GBD 2023 Disease and Injury and Risk Factor Collaborators (2025). Burden of 375 diseases and injuries, risk-attributable burden of 88 risk factors, and healthy life expectancy in 204 countries and territories, including 660 subnational locations, 1990–2023: A systematic analysis for the Global Burden of Disease Study 2023. Lancet.

[3] Jayawardena R., Byrne N.M., Soares M.J., Katulanda P., Hills A.P. (2015). The obesity epidemic in Sri Lanka revisited. Asia Pac. J. Public Health.

[4] Somasundaram N., Ranathunga I., Gunawardana K., Ahamed M., Ediriweera D., Antonypillai C.N., Kalupahana N. (2019). High Prevalence of Overweight/Obesity in Urban Sri Lanka: Findings from the Colombo Urban Study. J. Diabetes Res..

[5] Katulanda P., Jayawardena M.A., Sheriff M.H., Matthews D.R. (2011). Derivation of anthropometric cut-off levels to define CVD risk in Sri Lankan adults. Br. J. Nutr..

[6] Misra A., Shrivastava U. (2013). Obesity and dyslipidemia in South Asians. Nutrients.

[7] Riaz M., Lodhi S. (2025). Beyond BMI: Exploring obesity trends in the south Asian region. Obes. Pillars.

[8] Ahmed S.K., Mohammed R.A. (2025). Obesity: Prevalence, causes, consequences, management, preventive strategies and future research directions. Metabol. Open.

[9] Cao Y.J., Wang H.J., Zhang B., Qi S.F., Mi Y.J., Pan X.B., Wang C., Tian Q.B. (2020). Associations of fat and carbohydrate intake with becoming overweight and obese: An 11-year longitudinal cohort study. Br. J. Nutr..

[10] Hooper L., Abdelhamid A., Moore H.J., Douthwaite W., Skeaff C.M., Summerbell C.D. (2012). Effect of reducing total fat intake on body weight: Systematic review and meta-analysis of randomised controlled trials and cohort studies. BMJ.

[11] Liu X., Li Y., Tobias D.K., Wang D.D., Manson J.E., Willett W.C., Hu F.B. (2018). Changes in Types of Dietary Fats Influence Long-term Weight Change in US Women and Men. J. Nutr..

[12] Skilton M.R., Laville M., Cust A.E., Moulin P., Bonnet F. (2008). The association between dietary macronutrient intake and the prevalence of the metabolic syndrome. Br. J. Nutr..

[13] Ahluwalia N., Ferrieres J., Dallongeville J., Simon C., Ducimetiere P., Amouyel P., Arveiler D., Ruidavets J.B. (2009). Association of macronutrient intake patterns with being overweight in a population-based random sample of men in France. Diabetes Metab..

[14] Abete I., Astrup A., Martinez J.A., Thorsdottir I., Zulet M.A. (2010). Obesity and the metabolic syndrome: Role of different dietary macronutrient distribution patterns and specific nutritional components on weight loss and maintenance. Nutr. Rev..

[15] Ludwig D.S., Ebbeling C.B. (2018). The Carbohydrate-Insulin Model of Obesity: Beyond “Calories In, Calories Out”. JAMA Intern. Med..

[16] Bandara S., Kumara T., Dharmadasa S., Samaraweera R. (2021). Changes in Food Consumption Patterns in Sri Lanka: Food Security and Sustainability: A Review of Literature. Open J. Soc. Sci..

[17] Weerahewa J., Gedara P., Wijetunga C. (2018). Nutrition transition in Sri Lanka: A diagnosis. Ann. Nutr. Food Sci..

[18] Jayawardena R., Thennakoon S., Byrne N., Soares M., Katulanda P., Hills A. (2014). Energy and nutrient intakes among Sri Lankan adults. Int. Arch. Med..

[19] Weerasekara P.C., Withanachchi C.R., Ginigaddara G.A.S., Ploeger A. (2020). Understanding Dietary Diversity, Dietary Practices and Changes in Food Patterns in Marginalised Societies in Sri Lanka. Foods.

[20] Haq A., Maharani N., Mahati E., Winarni T.I., Pramono A. (2026). The diet–gene interaction: FTO rs9939609 and its role in obesity and metabolic syndrome among Asian populations. Egypt. J. Med. Hum. Genet..

[21] Bineid M.M., Ventura E.F., Samidoust A., Radha V., Anjana R.M., Sudha V., Walton G.E., Mohan V., Vimaleswaran K.S. (2025). A Systematic Review of the Effect of Gene-Lifestyle Interactions on Metabolic-Disease-Related Traits in South Asian Populations. Nutr. Rev..

[22] Sekar P., Lovegrove J.A., Surendran S., Vimaleswaran K.S. (2025). High Polyunsaturated Fatty Acid Intake Attenuates the Genetic Risk of Higher Waist Circumference in a Sri Lankan Adult Population. Nutrients.

[23] Surendran S., Alsulami S., Lankeshwara R., Jayawardena R., Wetthasinghe K., Sarkar S., Ellahi B., Lovegrove J.A., Anthony D.J., Vimaleswaran K.S. (2019). A genetic approach to examine the relationship between vitamin B12 status and metabolic traits in a South Asian population. Int. J. Diabetes Dev. Ctries..

[24] Huls A., Kramer U., Carlsten C., Schikowski T., Ickstadt K., Schwender H. (2017). Comparison of weighting approaches for genetic risk scores in gene-environment interaction studies. BMC Genet..

[25] Aschard H. (2016). A perspective on interaction effects in genetic association studies. Genet. Epidemiol..

[26] Ramya K., Radha V., Ghosh S., Majumder P.P., Mohan V. (2011). Genetic variations in the FTO gene are associated with type 2 diabetes and obesity in south Indians (CURES-79). Diabetes Technol. Ther..

[27] Chauhan G., Tabassum R., Mahajan A., Dwivedi O.P., Mahendran Y., Kaur I., Nigam S., Dubey H., Varma B., Madhu S.V. (2011). Common variants of FTO and the risk of obesity and type 2 diabetes in Indians. J. Hum. Genet..

[28] Scuteri A., Sanna S., Chen W.M., Uda M., Albai G., Strait J., Najjar S., Nagaraja R., Orru M., Usala G. (2007). Genome-wide association scan shows genetic variants in the FTO gene are associated with obesity-related traits. PLoS Genet..

[29] Vimaleswaran K.S., Bodhini D., Lakshmipriya N., Ramya K., Anjana R.M., Sudha V., Lovegrove J.A., Kinra S., Mohan V., Radha V. (2016). Interaction between FTO gene variants and lifestyle factors on metabolic traits in an Asian Indian population. Nutr. Metab..

[30] Mahajan A., Taliun D., Thurner M., Robertson N.R., Torres J.M., Rayner N.W., Payne A.J., Steinthorsdottir V., Scott R.A., Grarup N. (2018). Fine-mapping type 2 diabetes loci to single-variant resolution using high-density imputation and islet-specific epigenome maps. Nat. Genet..

[31] Grant S.F., Thorleifsson G., Reynisdottir I., Benediktsson R., Manolescu A., Sainz J., Helgason A., Stefansson H., Emilsson V., Helgadottir A. (2006). Variant of transcription factor 7-like 2 (TCF7L2) gene confers risk of type 2 diabetes. Nat. Genet..

[32] Scott L.J., Mohlke K.L., Bonnycastle L.L., Willer C.J., Li Y., Duren W.L., Erdos M.R., Stringham H.M., Chines P.S., Jackson A.U. (2007). A genome-wide association study of type 2 diabetes in Finns detects multiple susceptibility variants. Science.

[33] Sanghera D.K., Nath S.K., Ortega L., Gambarelli M., Kim-Howard X., Singh J.R., Ralhan S.K., Wander G.S., Mehra N.K., Mulvihill J.J. (2008). TCF7L2 polymorphisms are associated with type 2 diabetes in Khatri Sikhs from North India: Genetic variation affects lipid levels. Ann. Hum. Genet..

[34] Noordam R., Zwetsloot C.P.A., de Mutsert R., Mook-Kanamori D.O., Lamb H.J., de Roos A., de Koning E.J.P., Rosendaal F.R., Willems van Dijk K., van Heemst D. (2018). Interrelationship of the rs7903146 TCF7L2 gene variant with measures of glucose metabolism and adiposity: The NEO study. Nutr. Metab. Cardiovasc. Dis..

[35] Grau K., Cauchi S., Holst C., Astrup A., Martinez J.A., Saris W.H., Blaak E.E., Oppert J.M., Arner P., Rossner S. (2010). TCF7L2 rs7903146-macronutrient interaction in obese individuals’ responses to a 10-wk randomized hypoenergetic diet. Am. J. Clin. Nutr..

[36] Jayawardena R., Herath M.P. (2017). Development of a food atlas for Sri Lankan adults. BMC Nutr..

[37] WHO Expert Consultation (2004). Appropriate body-mass index for Asian populations and its implications for policy and intervention strategies. Lancet.

[38] Alberti K.G., Zimmet P., Shaw J., Group I.D.F.E.T.F.C. (2005). The metabolic syndrome—A new worldwide definition. Lancet.

[39] Sekikawa A., Shin C., Masaki K.H., Barinas-Mitchell E.J., Hirooka N., Willcox B.J., Choo J., White J., Evans R.W., Fujiyoshi A. (2013). Association of total marine fatty acids, eicosapentaenoic and docosahexaenoic acids, with aortic stiffness in Koreans, whites, and Japanese Americans. Am. J. Hypertens..

[40] West R.M. (2022). Best practice in statistics: The use of log transformation. Ann. Clin. Biochem..

[41] Ghasemi A., Zahediasl S. (2012). Normality tests for statistical analysis: A guide for non-statisticians. Int. J. Endocrinol. Metab..

[42] Loos R.J., Yeo G.S. (2014). The bigger picture of FTO: The first GWAS-identified obesity gene. Nat. Rev. Endocrinol..

[43] Peng S., Zhu Y., Xu F., Ren X., Li X., Lai M. (2011). FTO gene polymorphisms and obesity risk: A meta-analysis. BMC Med..

[44] Do R., Bailey S.D., Desbiens K., Belisle A., Montpetit A., Bouchard C., Pérusse L., Vohl M.-C., Engert J.C. (2008). Genetic variants of FTO influence adiposity, insulin sensitivity, leptin levels, and resting metabolic rate in the Quebec Family Study. Diabetes.

[45] Cheung C.Y., Tso A.W., Cheung B.M., Xu A., Ong K.L., Law L.S., Wat N.M., Janus E.D., Sham P.C., Lam K.S. (2011). Genetic variants associated with persistent central obesity and the metabolic syndrome in a 12-year longitudinal study. Eur. J. Endocrinol..

[46] Tan J.T., Dorajoo R., Seielstad M., Sim X.L., Ong R.T., Chia K.S., Wong T.Y., Saw S.M., Chew S.K., Aung T. (2008). FTO variants are associated with obesity in the Chinese and Malay populations in Singapore. Diabetes.

[47] Fawcett K.A., Barroso I. (2010). The genetics of obesity: FTO leads the way. Trends Genet..

[48] Zhang Z., Xu L., Xu X. (2021). The role of transcription factor 7-like 2 in metabolic disorders. Obes. Rev..

[49] Phillips C.M., Goumidi L., Bertrais S., Field M.R., McManus R., Hercberg S., Lairon D., Planells R., Roche H.M. (2012). Dietary saturated fat, gender and genetic variation at the TCF7L2 locus predict the development of metabolic syndrome. J. Nutr. Biochem..

[50] Palizban A., Rezaei M., Khanahmad H., Fazilati M. (2017). Transcription factor 7-like 2 polymorphism and context-specific risk of metabolic syndrome, type 2 diabetes, and dyslipidemia. J. Res. Med. Sci..

[51] Melzer D., Murray A., Hurst A.J., Weedon M.N., Bandinelli S., Corsi A.M., Ferrucci L., Paolisso G., Guralnik J.M., Frayling T.M. (2006). Effects of the diabetes linked TCF7L2 polymorphism in a representative older population. BMC Med..

[52] Hall K.D., Guo J. (2017). Obesity Energetics: Body Weight Regulation and the Effects of Diet Composition. Gastroenterology.

[53] Calton E.K., James A.P., Pannu P.K., Soares M.J. (2014). Certain dietary patterns are beneficial for the metabolic syndrome: Reviewing the evidence. Nutr. Res..

[54] MacInnis R.J., Hodge A.M., Dixon H.G., Peeters A., Johnson L.E., English D.R., Giles G.G. (2014). Predictors of increased body weight and waist circumference for middle-aged adults. Public Health Nutr..

[55] Paniagua J.A., Perez-Martinez P., Gjelstad I.M., Tierney A.C., Delgado-Lista J., Defoort C., Blaak E.E., Riserus U., Drevon C.A., Kiec-Wilk B. (2011). A low-fat high-carbohydrate diet supplemented with long-chain n-3 PUFA reduces the risk of the metabolic syndrome. Atherosclerosis.

[56] Kim H.N., Song S.W. (2019). Associations between Macronutrient Intakes and Obesity/Metabolic Risk Phenotypes: Findings of the Korean National Health and Nutrition Examination Survey. Nutrients.

[57] Bennett G., Bardon L.A., Gibney E.R. (2022). A Comparison of Dietary Patterns and Factors Influencing Food Choice among Ethnic Groups Living in One Locality: A Systematic Review. Nutrients.

[58] Anjana R.M., Sudha V., Abirami K., Gayathri R., Manasa V.S., Deepa M., Pradeepa R., Unnikrishnan R., Joshi S., Saboo B. (2025). Dietary profiles and associated metabolic risk factors in India from the ICMR-INDIAB survey-21. Nat. Med..

[59] Sartorius K., Sartorius B., Madiba T.E., Stefan C. (2018). Does high-carbohydrate intake lead to increased risk of obesity? A systematic review and meta-analysis. BMJ Open.

[60] Koyratty N., Nwabuikwi O., Silva R., Hess S.Y., Olney D.K. (2026). Diets, Fruit and Vegetable Intake, and Nutritional Status in Sri Lanka: A Scoping Review. Matern. Child. Nutr..

[61] San-Cristobal R., Navas-Carretero S., Martinez-Gonzalez M.A., Ordovas J.M., Martinez J.A. (2020). Contribution of macronutrients to obesity: Implications for precision nutrition. Nat. Rev. Endocrinol..

[62] Heianza Y., Qi L. (2017). Gene-Diet Interaction and Precision Nutrition in Obesity. Int. J. Mol. Sci..

[63] Czajkowski P., Adamska-Patruno E., Bauer W., Fiedorczuk J., Krasowska U., Moroz M., Gorska M., Kretowski A. (2020). The Impact of FTO Genetic Variants on Obesity and Its Metabolic Consequences is Dependent on Daily Macronutrient Intake. Nutrients.

[64] Labayen I., Ruiz J.R., Huybrechts I., Ortega F.B., Arenaza L., Gonzalez-Gross M., Widhalm K., Molnar D., Manios Y., DeHenauw S. (2016). Dietary fat intake modifies the influence of the FTO rs9939609 polymorphism on adiposity in adolescents: The HELENA cross-sectional study. Nutr. Metab. Cardiovasc. Dis..

[65] Sonestedt E., Roos C., Gullberg B., Ericson U., Wirfalt E., Orho-Melander M. (2009). Fat and carbohydrate intake modify the association between genetic variation in the FTO genotype and obesity. Am. J. Clin. Nutr..

[66] Celis-Morales C.A., Lyall D.M., Gray S.R., Steell L., Anderson J., Iliodromiti S., Welsh P., Guo Y., Petermann F., Mackay D.F. (2017). Dietary fat and total energy intake modifies the association of genetic profile risk score on obesity: Evidence from 48 170 UK Biobank participants. Int. J. Obes..

[67] Mattei J., Qi Q., Hu F.B., Sacks F.M., Qi L. (2012). TCF7L2 genetic variants modulate the effect of dietary fat intake on changes in body composition during a weight-loss intervention. Am. J. Clin. Nutr..

[68] Lottenberg A.M., Afonso Mda S., Lavrador M.S., Machado R.M., Nakandakare E.R. (2012). The role of dietary fatty acids in the pathology of metabolic syndrome. J. Nutr. Biochem..

[69] Wali J.A., Jarzebska N., Raubenheimer D., Simpson S.J., Rodionov R.N., O’Sullivan J.F. (2020). Cardio-Metabolic Effects of High-Fat Diets and Their Underlying Mechanisms-A Narrative Review. Nutrients.

[70] Forde C.G., de Graaf K. (2022). Influence of Sensory Properties in Moderating Eating Behaviors and Food Intake. Front. Nutr..

[71] Crovesy L., Rosado E.L. (2019). Interaction between genes involved in energy intake regulation and diet in obesity. Nutrition.

[72] Grau K., Hansen T., Holst C., Astrup A., Saris W.H., Arner P., Rossner S., Macdonald I., Polak J., Oppert J.M. (2009). Macronutrient-specific effect of FTO rs9939609 in response to a 10-week randomized hypo-energetic diet among obese Europeans. Int. J. Obes..

[73] Chen X., Ayala I., Shannon C., Fourcaudot M., Acharya N.K., Jenkinson C.P., Heikkinen S., Norton L. (2018). The Diabetes Gene and Wnt Pathway Effector TCF7L2 Regulates Adipocyte Development and Function. Diabetes.

[74] Bauer W., Adamska-Patruno E., Krasowska U., Moroz M., Fiedorczuk J., Czajkowski P., Bielska D., Gorska M., Kretowski A. (2021). Dietary Macronutrient Intake May Influence the Effects of TCF7L2 rs7901695 Genetic Variants on Glucose Homeostasis and Obesity-Related Parameters: A Cross-Sectional Population-Based Study. Nutrients.

[75] Nienaber-Rousseau C. (2025). Understanding and applying gene-environment interactions: A guide for nutrition professionals with an emphasis on integration in African research settings. Nutr. Rev..

[76] Ravelli M.N., Schoeller D.A. (2020). Traditional Self-Reported Dietary Instruments Are Prone to Inaccuracies and New Approaches Are Needed. Front. Nutr..

