# Supplementary material for: Interactions Between Genetic Risk and Macronutrient Intake on Obesity-Related Traits in Sri Lankan Adults
Source: Nutrients. 2026 Sep 9;18(18):2951. doi: 10.3390/nu18182951 (PMC13610610; doi:10.3390/nu18182951)
Supplement: Supplementary file 1 [file nutrients-18-02951-s001.zip › Bineid et al_Supplement Table S2.pdf]

Table S2. Associations of *FTO* rs8050136 and rs1588413, *TCF7L2* SNPs rs7903146 with obesity traits (additive model)

		***FTO* rs8050136**		
	**Mean ± SD**	**Mean ± SD**	**Mean ± SD**	***p* value**
	**(CC) (n=160)**	**(CA) (n=190)**	**(AA) (n=48)**	
BMI (kg/m^2)	23.95 ± 3.56	24.95 ± 3.8	25.43 ±3.7	0.057
WC (cm)	85.29 ± 10.3	86.42 ± 9.8	87.62 ± 9.5	0.250
		***FTO* rs1588413**		
	**(GG) (n=225)**	**(GA) (n=139)**	**(AA) (n=34)**	
BMI (kg/m^2)	24.43 ± 3.8	24.12 ± 3.5	24.62 ± 3.8	0.698
WC (cm)	85.82 ± 9.8	85.89 ± 10.1	88.88 ± 10.2	0.233
		***TCF7L2* rs7903146**		
	**(CC) (n=200)**	**(CT) (n=170)**	**(TT) (n=28)**	
BMI (kg/m^2)	24.31 ± 3.5	24.43 ± 4	23.95 ± 3.6	0.878
WC (cm)	86.07 ± 8.9	86.22 ± 10.7	85. 64 ± 12.2	0.982

Data presented as mean ± SD. *P* values obtained using GLM.
p values are adjusted for age and sex.
