# Supplementary material for: Interactions Between Genetic Risk and Macronutrient Intake on Obesity-Related Traits in Sri Lankan Adults
Source: Nutrients. 2026 Sep 9;18(18):2951. doi: 10.3390/nu18182951 (PMC13610610; doi:10.3390/nu18182951)
Supplement: Supplementary file 1 [file nutrients-18-02951-s001.zip › Bineid et al_Supplement Table S4.pdf]

Table S4. Linear regression models examining GRS–macronutrient interactions on continuous obesity-related outcomes.

Obesity outcomes	GRS*Fat (%)		GRS*Carbohydrates (%)		GRS*Protein (%)	
	B (95% CI)	*p* Value	B (95% CI)	*p* Value	B (95% CI)	*p* Value
BMI	-0.003 (-0.005-0.001)	**0.038**	0.003 (0.001-0.005)	**0.010**	0.0001 (0.-0.003-0.002)	0.986
WC	-0.001 (-0.003-0.001)	0.111	0.002 (0.001-0.003)	**0.038**	0.001 (-0.002-0.002)	0.732

$P_{interaction}$ values were obtained using a linear regression using log-transformed outcome variables. Model adjusted for age, sex, and total energy intake. Values in bold represent statistical significance ($p < 0.05$). **Abbreviations**: BMI, body mass index; WC, waist circumference; GRS, genetic risk score.
