# Supplementary material for: Interactions Between Genetic Risk and Macronutrient Intake on Obesity-Related Traits in Sri Lankan Adults
Source: Nutrients. 2026 Sep 9;18(18):2951. doi: 10.3390/nu18182951 (PMC13610610; doi:10.3390/nu18182951)
Supplement: Supplementary file 1 [file nutrients-18-02951-s001.zip › Bineid et al_Supplement_Table S1.pdf]

Table S1. Genotypes and allele frequency of selected SNPs

Gene	SNP	Chromosome location	Location of SNP	Nucleotide change	Genotype Count	Allele Frequency (%)	Overall MAF	HWE P-value
FTO	rs8050136	16:53782363	Intronic	C/A	CC=160<br>CA=190<br>AA=48	C=64<br>A=36	0.36	0.46
FTO	rs1588413	16:54115620	3 Prime UTR Variant	G/A	GG=225<br>AG=139<br>AA=34	G=74<br>A=26	0.26	0.06
TCF7L2	rs7903146	10:112998590	Intronic	C/T	CC=200<br>TC=170<br>TT=28	C=72<br>T=28	0.28	0.31

Abbreviations: SNP, single-nucleotide polymorphism; *TCF7L2*, transcription factor 7-like2; *FTO*, Fat mass and obesity associate
