# Supplementary material for: Interactions Between Genetic Risk and Macronutrient Intake on Obesity-Related Traits in Sri Lankan Adults
Source: Nutrients. 2026 Sep 9;18(18):2951. doi: 10.3390/nu18182951 (PMC13610610; doi:10.3390/nu18182951)
Supplement: Supplementary file 1 [file nutrients-18-02951-s001.zip › Bineid et al_Supplement_Table S3.pdf]

Table S3. Baseline characteristics of the study population by GRS groups (n = 398)

	n	Mean	SD	Low GRS (<2 risk allele)			High GRS (≥2 risk alleles)			*p*-value
				n	Mean	SD	n	Mean	SD	
Age	398	42.52	8.3	165	43.11	8.49	233	42.09	8.15	0.257*
Weight (kg)	398	64.12	12.07	165	62.18	11.28	233	65.49	12.44	**0.008***
WC (cm)	398	86.11	9.99	165	84.98	9.38	233	86.91	10.35	0.075*
BMI	398	24.34	3.74	165	23.94	3.54	233	24.61	3.86	0.090*
Energy (kcal)	398	1898.61	569.54	165	1915.33	575.85	233	1886.68	565.96	0.631*
Carbohydrate (energy%)	398	63.44	6.1	165	63.71	5.95	233	63.24	6.21	0.448
Fat (energy %)	398	24.95	5.48	165	24.81	5.56	233	25.05	5.43	0.674
Protein (energy %)	398	12.28	5.4	165	12.19	5.34	233	12.34	5.45	0774
Fibre (g)	398	8.81	3.57	165	8.64	3.63	233	8.92	3.52	0.330*
Increased BMI (%)	398	63.6		99	39.1		154	60.9		0.213†
Increased WC (%)	398	52.8		77	36.7		133	63.3		**0.040†**

Data are presented as mean and SD for continuous variables and percentages for categorical variables. *p*-values for continuous variables were derived using an independent-sample T-test. *Analyses were conducted on log-transformed variables. †*p*-values for categorical variables were obtained by a Chi-square test. Values in bold represent statistical significance ($p < 0.05$).

Abbreviations: WC, waist circumference; BMI, body mass index; GRS, genetic risk score.
